# Endoscopic submucosal dissection of uncommon huge gastric pyloric gland adenoma

**DOI:** 10.1055/a-2667-7474

**Published:** 2025-08-20

**Authors:** Ahmed Altonbary, Asmaa Gameel, Hazem Hakim, Khaled Zalata

**Affiliations:** 1 68780 Department of Gastroenterology and Hepatology, Mansoura University, Mansoura, Egypt; 2 68780 Department of Pathology, Mansoura University, Mansoura, Egypt

Pyloric gland adenoma (PGA) is a rare gastrointestinal tumor that is characterized by dense packing of gastric pyloric glands with occasional cystic dilatation 1 . PGAs represent almost 2.7% of all gastric polyps with a conversion rate of 47% to invasive adenocarcinoma 2 . There are currently no evidence-based recommendations on resection technique and surveillance protocol specific for PGAs. Previously reported large gastric PGA was treated with laparoscopic resection 1 . However, endoscopic submucosal dissection (ESD) has been recently used in the treatment of smaller PGAs 3 4 . Herein, we report a rare case of huge gastric PGA treated with ESD.

A 68-year-old female came to our hospital with complaints of recurrent abdominal pain and vomiting. Laboratory investigations were unremarkable apart from iron deficiency anemia. Gastroscopy revealed a huge polypoidal mass with a thick stalk arising from the anterior gastric wall and occupying the fundus. Biopsy suggested PGA with low-grade dysplasia. The colonoscopy was unremarkable. Subsequently, abdominal computed tomography showed an intragastric polypoidal mass occupying the lumen with no extra-gastric extension or lymph nodes. The lesion was removed by ESD using a 3-mm ball-type knife Endocut Q, effect 2, for incision, forced coagulation 4 for dissection, and soft coagulation 5 using coagulation grasper for hemostasis, followed by resection bed closure with 5 endoclips (17 mm) with no intraprocedural complications ( Video 1 ). The resected specimen was retrieved in piecemeal and measured about 13 cm × 9 cm ( Fig. 1 ). Pathological examination of the lesion revealed tubular adenomatous proliferation with packed and dilated pyloric glands with ground glass cytoplasm lined and covered with mildly atypical cells with low mitotic activity consistent with PGA with low-grade dysplasia and clear deep resection margin ( Fig. 2 ). No adverse events were reported after the procedure. The patient was discharged after 1 day and scheduled for follow-up gastroscopy after 3 months.

Endoscopic submucosal dissection of an uncommon huge gastric pyloric gland adenoma.Video 1

**Fig. 1:**
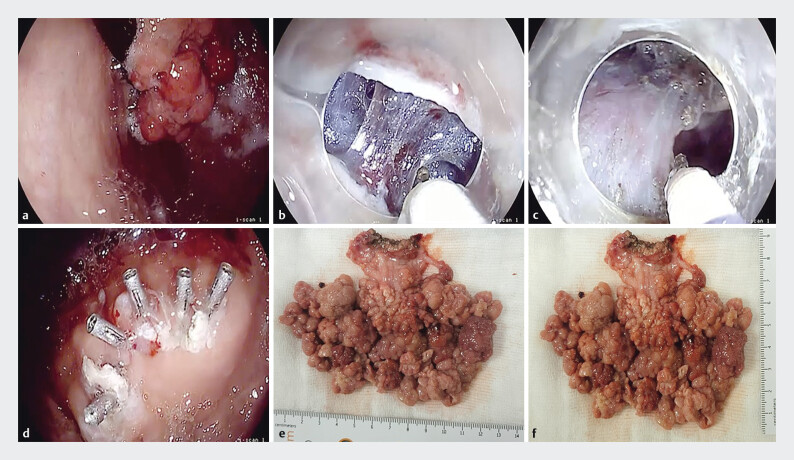
Endoscopic submucosal dissection of gastric pyloric gland adenoma: **a** polypoidal mass with thick stalk occupying the fundus, **b** stalk incision with 3-mm ball-type knife, **c** dissection with forced coagulation, **d** resection bed closure with 5 endoclips, and **e, f** the resected specimen measured about 13 cm × 9 cm.

**Fig. 2:**
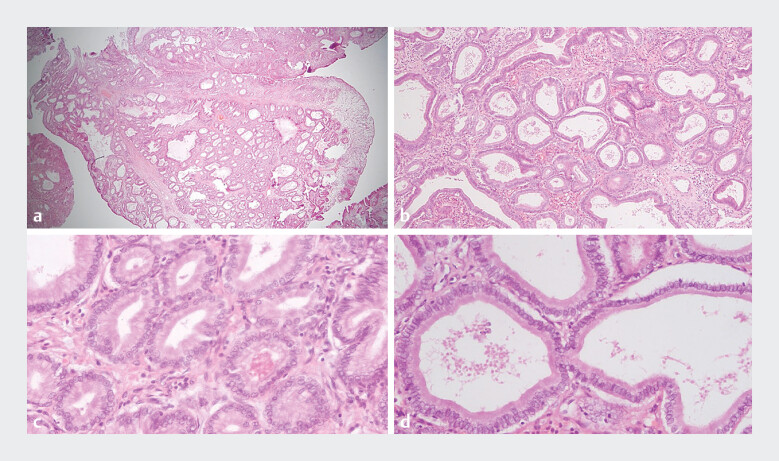
Pathological examination of the retrieved specimen: **a, b** tubular adenomatous proliferation with packed and dilated pyloric glands, **c** lined and covered with mildly atypical cells, and **d** ground glass cytoplasm, consistent with PGA with low-grade dysplasia.

Endoscopy_UCTN_Code_CCL_1AB_2AD_3AB

## Notes

Endoscopy E-Videos https://eref.thieme.de/e-videos
*E-Videos* is an open access online section of the journal *Endoscopy* , reporting on interesting cases and new techniques in gastroenterological endoscopy. All papers include a high-quality video and are published with a Creative Commons CC-BY license. Endoscopy E-Videos qualify for HINARI discounts and waivers and eligibility is automatically checked during the submission process. We grant 100% waivers to articles whose corresponding authors are based in Group A countries and 50% waivers to those who are based in Group B countries as classified by Research4Life (see: https://www.research4life.org/access/eligibility/ ). This section has its own submission website at https://mc.manuscriptcentral.com/e-videos .
